# Expanding the neuroimmune research toolkit with *in vivo* brain organoid technologies

**DOI:** 10.1242/dmm.052200

**Published:** 2025-04-15

**Authors:** Ai Tian, Afrin Bhattacharya, Julien Muffat, Yun Li

**Affiliations:** ^1^ Program in Neurosciences and Mental Health, The Hospital for Sick Children, 686 Bay Street, Toronto, ON M5G 0A4, Canada; ^2^ Department of Molecular Genetics, University of Toronto, 1 King's College Circle, Toronto, ON M5S 1A8, Canada; ^3^ Program in Developmental and Stem Cell Biology, The Hospital for Sick Children, 686 Bay Street, Toronto, ON M5G 0A4, Canada

## Abstract

Human pluripotent stem cell-derived microglia-like cells (MLCs) and brain organoid systems have revolutionized the study of neuroimmune interactions, providing new opportunities to model human-specific brain development and disease. Over the past decade, advances in protocol design have improved the fidelity, reproducibility and scalability of MLC and brain organoid generation. Co-culturing of MLCs and brain organoids have enabled direct investigations of human microglial interactions *in vitro*, although opportunities remain to improve microglial maturation and long-term survival. To address these limitations, innovative xenotransplantation approaches have introduced MLCs, organoids or neuroimmune organoids into the rodent brain, providing a vascularized environment that supports prolonged development and potential behavioral readouts. These expanding *in vitro* and *in vivo* toolkits offer complementary strategies to study neuroimmune interactions in health and disease. In this Perspective, we discuss the strengths, limitations and synergies of these models, highlighting important considerations for their future applications.

## Teaser

**Summary:** This Perspective examines diverse neuroimmune research platforms, highlighting recent advances in *in vivo* xenotransplantation alongside alternative techniques, discussing their complementary benefits, limitations and potential for advancing the field.

## Introduction

### “All models are wrong, but some are useful.”

Understanding the complexity of the human brain and its disorders requires advanced models that capture realistic interactions between neurons, astrocytes, oligodendrocytes and microglia. These cells form a highly structured parenchyma (Barres, 2008; Masland, 2004; Siletti et al., 2023), traversed by blood vessels and cerebrospinal fluid-filled ventricles (Rasmussen et al., 2022; Walchli et al., 2024). Together, these different components enable essential brain functions, from physiological homeostasis to higher cognition. Neuroimmune interactions, particularly those involving microglia, play essential roles in maintaining brain health, influencing neurogenesis, synaptic pruning, myelination and brain integrity (Lawrence et al., 2024; McNamara et al., 2023; Sierra et al., 2019). Their dysfunction is increasingly linked to neurological disorders including autism spectrum disorder (ASD) and Alzheimer's disease (Salter and Stevens, 2017). Understanding the interaction of microglia with other brain cells in both healthy and diseased contexts is thus critical for developing targeted therapies.

Human pluripotent stem cell (hPSC)-derived brain organoids have transformed the study of neurodevelopment and disease (Bhattacharya et al., 2022; Tian et al., 2020; Zhang et al., 2023). These three-dimensional *in vitro* neural tissues offer a powerful alternative to human brain studies, overcoming ethical and logistical constraints. They allow extensive *in vitro* experimentation, including genetic manipulation and long-term observation (Eichmuller and Knoblich, 2022). Although neurons and macroglia (astrocytes and oligodendrocytes) develop naturally in brain organoids owing to their neuroectodermal lineage, microglia are typically absent because they are derived from the mesoderm (Ginhoux et al., 2010). To address this, we and others have developed protocols to differentiate hPSCs into microglia-like cells (MLCs) and incorporate these MLCs into neural organoids, establishing neuroimmune organoids that enable the study of neuro-microglial interactions *in vitro* (Abud et al., 2017; Cakir et al., 2022; Fagerlund et al., 2021; Lin et al., 2018; Muffat et al., 2018, 2016; Ormel et al., 2018; Park et al., 2023; Popova et al., 2021; Sabate-Soler et al., 2022; Xu et al., 2021).

**Fig. 1.**
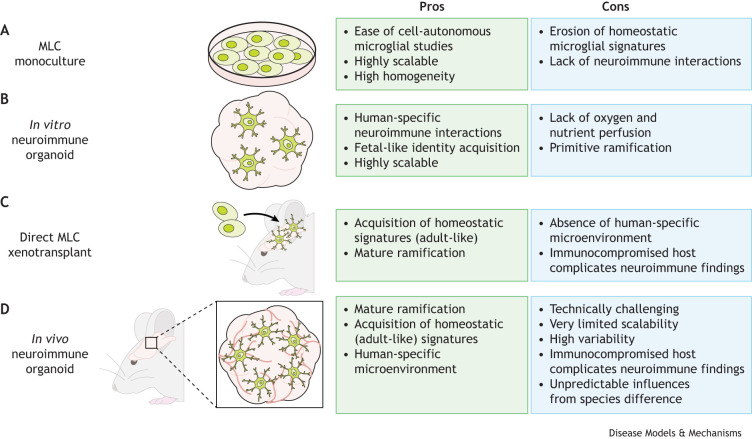
**Schematic of different methods for studying human pluripotent stem cell-derived microglia and summary of advantages and limitations.** (A) Monocultured microglia-like cells (MLCs). (B) Microglia co-cultured with brain organoids to generate *in vitro* neuroimmune organoids. (C) Direct xenotransplantation of MLCs into the brain of an animal host. (D) Neuroimmune brain organoids xenotransplanted into the brain of an animal host.

Microglia are highly dynamic immune cells that continuously adapt to environmental changes throughout development, injury, disease and aging, thus supporting homeostasis in the brain (Bennett et al., 2018; Gosselin et al., 2017; Martins-Ferreira et al., 2025). Originating from the yolk sac, they migrate into the brain and adopt diverse cellular states (Bian et al., 2020; Ginhoux et al., 2010; Kracht et al., 2020; Paolicelli et al., 2022). Towards adulthood, microglia remain heterogeneous but primarily acquire a base identity, dominated by homeostatic transcriptional profiles, ramified (branched) morphology and physiologically relevant functions such as immune surveillance (Butovsky and Weiner, 2018; Davalos et al., 2005; Kettenmann et al., 2011; Nimmerjahn et al., 2005). This basal state is rapidly lost in microglia extracted from the brain, indicating that microglial state is tightly regulated by the brain's microenvironment (Bohlen et al., 2017; Gosselin et al., 2017). Accordingly, MLCs cultured alone exhibit immature morphology and have limited expression of key homeostatic markers (Fig. 1A). In contrast, when co-cultured within brain organoids and equivalent 3D cultures, MLCs show improved morphology, including ramification (Abud et al., 2017; Fagerlund et al., 2021; Muffat et al., 2018, 2016), surveillance and injury response (Haenseler et al., 2017; Muffat et al., 2016; Park et al., 2023; Popova et al., 2021), as well as expression of homeostatic markers such as purinergic receptor P2Y12 (*P2RY12*) and transmembrane protein 119 (*TMEM119*) (Park et al., 2023; Popova et al., 2021; Xu et al., 2021). Together, these findings suggest that an organotypic microenvironment promotes programming toward a homeostatic microglial cell fate (Fig. 1B, Table 1) (Jin et al., 2022; Popova et al., 2021).

**Table 1.** Comparison of human *in vitro* neuroimmune organoid and microglia xenotransplantation studies

Reference	Microglia source	Organoid protocol/host mice*	Microglial morphology and motility reported	Latest timepoint assessed (post co-culture)	Homeostatic microglia markers examined	Notable phenotypes	GEO accession number/link
*In vitro* neuroimmune organoid							
Muffat et al., 2016	hPSC-derived microglia	Organotypic 3D neuroglial cultures	Ramified; surveillance (response to focal injury)	14 days	P2RY12, TMEM119 (RNA and IHC), prior to co-culture	• Microglia branching, surveillance and chemotaxis	GSE85839
Muffat et al., 2018	hPSC-derived microglia	Neural progenitor aggregate	Ramified; morphology change induced by infection	7 days	N/A	• Response of microglia to zika virus infection	
Abud et al., 2017	hPSC-derived microglia	Unguided brain organoid	Ramified (varying degrees)	7 days	*P2RY12*, *CX3CR1* (RNA), prior to co-culture	• Motile and morphological response to needle injury • Tiling of microglia	GSE89189
Ormel et al., 2018	Innate	Unguided brain organoid	Rounded initially; progressive ramification	119 days	*P2RY12*, *TMEM119* (RNA)	• Response to inflammatory stimulus (LPS and dexamethasone)	GSE102335
Lin et al., 2018	hPSC-derived microglia	Cortical organoid	Ramified; surveillance behavior	30 days	RNA, prior to co-culture	• APOE4 mutant microglia show morphology change and defective β-amyloid clearance in organoids	GSE102956
Popova et al., 2021	Human fetal microglia; hPSC-derived microglia	Cortical organoid	Amoeboid and ramified; motile processes	35 days	Homeostatic signature (*P2RY12*, *CX3CR1*, *P2RY13*, *VSIR*) increased in co-culture (RNA)	• Decreased cell stress in organoids with microglia • Increased neural network synchronicity	GSE180945
Fagerlund et al., 2021	hPSC-derived microglia	Unguided brain organoid	Intermediate, rod-shaped, spheric; progressive ramification	183 days	N/A	• Increased neuronal maturation • Spontaneous network activity	
Xu et al., 2021	hPSC-derived microglia	Neural progenitor aggregate	Ramified	69 days	TMEM119 (IHC)	• Synaptic pruning • Phagocytic activity • Response to zika virus infection	
Sabate-Soler et al., 2022	hPSC-derived microglia	Midbrain organoid	Ramified, round, elongated	70 days	P2RY12, TMEM119 (IHC) prior to co-culture	• Reduced cell death and oxidative stress following co-culture • Synapse remodeling	https://webdav.lcsb.uni.lu/public/data/cx25-ht49/
Jin et al., 2022	hPSC-derived microglia	Neural progenitor aggregate	Low ramification	56 days	TMEM119 (IHC)	• Down syndrome microglia show increased synaptic pruning and reactive morphology in organoids	GSE189227
Cakir et al., 2022	Innate	Overexpression of PU.1 in cortical organoids	Amoeboid to progressive partial ramification; motile behavior	∼100 days	P2RY12, TMEM119 (IHC, RNA)	• Microglia protected organoids from β-amyloid (reduced expression of apoptosis, ferroptosis, AD-associated genes) • Pooled CRISPRi screen targeting AD genes	GSE175722
Park et al., 2023	hPSC-derived microglia	Unguided brain organoid	Amoeboid; morphological change induced by laser injury	30 days	*P2RY12*, *CX3CR1*, *SALL1* increased in organoids, but not *TMEM119* (RNA)	• Decreased organoid size mediated by lipid transfer • Increased neuronal maturation • Increased ER stress, hypoxia response	GSE242894 GSE241127
Direct xenotransplantation of microglia							
Abud et al., 2017	hPSC-derived microglia	MITRG mice (hCSF1; hIL3/hGM-CSF; hTPO); Rag 5xfAD mice	Highly branched morphology	8 weeks	P2RY12, TMEM119 (IHC)	• Engraftment and long-term survival • Highly ramified morphology and high P2RY12 expression suggested active surveillance • Migrate and extend processes towards β-amyloid plaques • Phagocytosis of fibrillar β-amyloid	
Hasselmann et al., 2019	hPSC-derived microglia	MITRG mice (hCSF1; hGM-CSF; hTPO or hCSF1 only)	Complex ramified morphology; motile response to laser ablation	8 weeks	P2RY12, TMEM119 (IHC); *P2RY12*, *TMEM119*, *SALL1* (RNA)	• Respond to local injury (focal laser ablation), brain trauma, systemic inflammatory challenge (LPS) and β-amyloid plaques	GSE133434
Mancuso et al., 2019	hPSC-derived microglia	*Rag2*^−/−^*Il2rg*^−/−^ hCSF1KI mice+CSF1R inhibitor	Complex ramified morphology	8 weeks	P2RY12, TMEM119 (IHC); *P2RY12*, *TMEM119*, *SALL1* (RNA)	• Express human-specific AD risk genes • Oligomeric β-amyloid induces a divergent response in human versus mouse microglia	GSE137444
Svoboda et al., 2019	hPSC-derived microglia	NSG-T (hIL3; hSCF; hGM-CSF); NSG-Q (hIL3; hSCF; hGM-CSF; hCSF1)	Ramified	120 days	P2RY12, TMEM119 (IHC); *P2RY12*, *SALL1* (RNA)	• Response to inflammatory stimulus (LPS)	GSE139194
Xu et al., 2020	hPSC-derived microglia	*Rag2*^−/−^*Il2rg*^−/−^ hCSF1KI mice	Complex ramified morphology	6 months	P2RY12, TMEM119 (IHC); *P2RY12*, *SALL1*, *TMEM119* (RNA)	• Dynamic response to cuprizone-induced demyelination • Species-specific transcriptomic differences in the expression of neurological disease-risk genes	GSE129178 GSE139161

*Mouse strains used for xenotransplantation were immunocompromised mice with human cytokines knocked in (except Rag 5xfAD mice, which are a model for AD).

AD, Alzheimer's disease; APOE4, apolipoprotein E4; CRISPRi, clustered regularly interspaced short palindromic repeats interference; CSF1, macrophage colony-stimulating factor 1; CSF1R, macrophage colony-stimulating factor 1 receptor; *CX3CR1*, CX3C motif chemokine receptor 1; ER, endoplasmic reticulum; GEO, Gene Expression Omnibus; GM-CSF, granulocyte-macrophage colony-stimulating factor (also known as CSF2); h, human; hPSC, human pluripotent stem cell; IHC, immunohistochemistry; IL, interleukin; LPS, lipopolysaccharide; N/A, not applicable; NSG, NOD scid gamma; PU.1, transcription factor PU.1 (also known as SPI1); P2RY, purinergic receptor P2Y; *SALL1*, spalt-like transcription factor 1; SCF, stem cell factor (also known as KITLG); TMEM119, transmembrane protein 119; TPO, thyroid peroxidase; VSIR, V-set immunoregulatory receptor; 3D, three-dimensional.

Despite these advances, *in vitro* neuroimmune organoids do not fully recapitulate mature microglial biology. Compared to postnatal or adult microglia, MLCs within organoids exhibit limited ramification and an incomplete transcriptional profile (Park et al., 2023; Popova et al., 2021). This suggests that the *in vitro* organoid environment lacks the critical features necessary for the continued maturation of MLCs, such as vasculature, relevant growth factors, electrophysiological inputs and long-term cellular viability, all of which are important for microglial development.

## Bringing *in vitro* systems *in vivo*

To provide a more physiological brain environment for MLCs, researchers have turned to xenotransplantation into the brains of immunocompromised rodents, demonstrating robust engraftment and long-term survival (Fig. 1C, Table 1) (Abud et al., 2017; Hasselmann et al., 2019; Mancuso et al., 2019; Svoboda et al., 2019; Xu et al., 2020). The grafted MLCs exhibit ramified branching patterns, surveillance behavior and gene signatures resembling adult homeostatic microglia, suggesting that host-derived cues enhance their maturation and fidelity. Separately, neural organoids without MLCs have been xenotransplanted into rodent models, aiming to improve oxygen and nutrient supply through host vascularization (Bhaduri et al., 2020; Cakir et al., 2019; Jgamadze et al., 2023; Mansour et al., 2018; Revah et al., 2022; Wang et al., 2024). The xenotransplanted organoids show reduced cell stress, improved cell fate specification, enhanced neuronal and glial maturation, host neural circuit integration and even host behavior modulation (Bhaduri et al., 2020; Jgamadze et al., 2023; Revah et al., 2022). Together, these findings underscore the importance of a physiological brain environment in promoting MLC and organoid maturation.

A recent study by Schafer et al. (2023) combined MLC and organoid xenotransplantation by grafting human neuroimmune organoids into the adult mouse brain. This approach represents a powerful tool for studying human neuroimmune interactions *in vivo* (Schafer et al., 2023) (Fig. 1D). Below, we highlight key insights gained from this study.

Xenotransplantation of neuroimmune organoids provides the best demonstration to date that hPSC-derived MLCs can achieve postnatal-like maturity while remaining embedded within a human brain envrionment.

### Recapitulating human postnatal microglial identity and morphology

Xenotransplantation of neuroimmune organoids provides the best demonstration to date that hPSC-derived MLCs can achieve postnatal-like maturity while remaining embedded within a human brain envrionment (Schafer et al., 2023). MLCs within transplanted organoids exhibited soma size and process complexity closely resembling that of mature microglia *in vivo* (Schafer et al., 2023; Schwabenland et al., 2021). Transcriptomic analysis revealed the stepwise acquisition of homeostatic identity, with progressive expression of microglia sensome genes, mirroring fetal-to-adult transitions observed *in vivo* (Han et al., 2023; Kracht et al., 2020; Matcovitch-Natan et al., 2016). In contrast, MLCs within *in vitro* organoids do not achieve this degree of morphometric and transcriptional maturity, potentially due to the limited maturity of the organoid microenvironment (Bhaduri et al., 2020; Uzquiano et al., 2022). Notably, MLCs in xenotransplanted neuroimmune organoids express human-specific genes absent in directly xenotransplanted MLCs, highlighting the importance of a human neural microenvironment in microglial identity acquisition (Schafer et al., 2023).

### Insights into disease progression

Neuroimmune organoid xenotransplantation offers exciting opportunities for longitudinal, organism-level investigations into microglial physiology and pathophysiology. In their proof-of-concept study, Schafer et al. (2023) examined human MLCs derived from individuals with ASD and neurotypical controls and observed reactive morphological phenotypes in xenotransplanted organoids, influenced by the ASD brain organoid environment in a non-cell-autonomous manner. However, it remains unclear whether such phenotypes can be recapitulated using *in vitro* neuroimmune organoid platforms.

Given the central role of microglia in late-onset disorders (Hickman et al., 2018; Romero-Molina et al., 2022), this platform would be particularly useful for studying long-term neuroimmune crosstalk in conditions such as Alzheimer's disease, demyelinating disorders and chronic inflammation. Although the approach itself relies on surgical resection and immunosuppression, it should facilitate studies of subsequent brain insults, as well as any efforts to perform therapeutic interventions (Jgamadze et al., 2023; Kitahara et al., 2020). Given that xenotransplanted neural organoids can integrate into host neural circuits and influence behavior, extending this research to neuroimmune organoids will provide unique opportunities to explore how microglia shape behavioral outcomes (Revah et al., 2022).

### Improving existing *in vitro* platforms

Another important insight from the Schafer et al. (2023) study is that the xenotransplanted organoid offered a more supportive environment for the resident human microglia. Unlike MLCs in direct xenotransplantation or *in vitro* neuroimmune organoids, which reportedly require human factors such as macrophage colony-stimulating factor 1 (CSF1) and interleukin (IL)34 for prolonged survival (Sehgal et al., 2021; Stanley and Chitu, 2014), MLCs within xenotransplanted neuroimmune organoids persisted without these supplements (Schafer et al., 2023). This suggests that host-derived influences – including vascularization, cytokine exchange and nutrient flow – enhance organoid fidelity, promoting MLC residence and maturation (Wang et al., 2023). These findings highlight a key avenue to refine *in vitro* neuroimmune models by identifying and incorporating the instructive factors derived from the host or organoid microenvironment.

## Limitations of the neuroimmune xenotransplantation approach

### Complexity and variability

Despite the significant advancements of the *in vivo* neuroimmune organoid model of Schafer et al. (2023), several challenges could hinder widespread adoption. Xenotransplantation requires technically demanding surgical procedures and expertise in working with immunocompromised mice under aseptic conditions. Factors such as graft size, survival, location, composition, vascularization and MLC density can vary significantly between experiments, complicating standardization and reproducibility (Chen et al., 2019; Pasca et al., 2024). Additionally, downstream analyses, such as observations of MLC motility and morphology, require invasive and sophisticated imaging techniques that are not readily accessible to most laboratories.

### Species differences and use of immunocompromised hosts

Another important consideration is the reliance on rodent hosts, which introduces species differences that may alter human cell behavior in unpredictable ways. Although the human organoid graft creates a niche dominated by human cellular influences that is absent in direct MLC xenotransplants, host cell migration and fluid transport make local environment influences difficult to evaluate (Schafer et al., 2023). Additionally, the heterochronic developmental tempo between rodent and human brains, as well as mismatched age between the organoid graft and the rodent brain, complicate interpretation (Semple et al., 2013). Alternative models such as xenotransplants into non-human primates or human cortical slices have been proposed, but raise additional ethical and practical considerations (Kitahara et al., 2020). Importantly, the use of immunocompromised rodents hinders studies on microglial response to infection and inflammation in the absence of a fully competent immunological environment, limiting experimental scope. This underscores a significant limitation of the *in vivo* organoid approach, particularly when addressing immunological questions.

### Scalability and cost

Scalability is another important limitation of the *in vivo* neuroimmune organoid model. Each organoid requires an individual living host, drastically restricting assay throughput and statistical power. Immunocompromised rodents are also costly to purchase and maintain, and the need for one animal per organoid further escalates costs, making this technology inaccessible to many laboratories.

[…] we encourage researchers to carefully consider the respective strengths and limitations of *in vivo* and *in vitro* models, to select the most appropriate approach based on experimental goals, available resources and biological questions.

## Benefits of existing *in vitro* neuroimmune platforms

Although several benefits of *in vivo* transplantation are clear, the value of *in vitro* systems for studying MLCs and neuroimmune interactions cannot be understated. Monoculture systems, for example, offer unique advantages for investigating cell-intrinsic microglial functions, allowing high-throughput studies of key macrophage staples such as phagocytosis and cytokine responses, making them invaluable for studying innate immunity (McQuade et al., 2020). Importantly, monoculture systems can be readily used to study the transitions between homeostatic and disease-relevant cellular states induced by pathologically relevant exposures (Fig. 1A) (Dolan et al., 2023).

*In vitro* neuroimmune organoids also provide a powerful platform for studying neuro-microglial interactions without xenotransplantation (Fig. 1B). Whereas Schafer et al. (2023) reported a rapid decline in MLC numbers and amoeboid morphology, other studies have demonstrated that MLCs within organoids can be maintained for weeks, displaying ramified branching and homeostatic marker expression (Fagerlund et al., 2021; Sabate-Soler et al., 2022; Xu et al., 2021). These *in vitro* neuroimmune organoids have enabled detailed studies of the influences of microglia on organoid development and vice versa, revealing pleiotropic MLC roles in synaptic pruning, neurogenesis regulation, electrophysiological maturation and cellular stress reduction (Table 1).

Importantly, both MLC monocultures and *in vitro* neuroimmune organoids have been applied to model various disease conditions, including Down syndrome, zika virus infection and β-amyloid plaque exposure in Alzheimer's disease (Drager et al., 2022; Garcia-Reitboeck et al., 2018; Konttinen et al., 2019) (Table 1). These studies highlight their value for analyzing pathological phenotypes while offering superior accessibility and scalability compared to xenotransplantation, enabling systematic phenotypic analyses in higher-throughput assays, such as genetic or drug screening. In particular, culture medium conditions can be controlled, enabling the study of systematic changes in gas tensions and biochemical compositions (Bohlen et al., 2017; Liu et al., 2025). These advantages make *in vitro* systems particularly well suited to large-scale studies that are not feasible with the xenotransplantation approach.

Finally, unlike xenotransplantation, *in vitro* systems provide an entirely human, isogenic and immunocompetent environment, eliminating untold confounding effects of species differences and reliance on immunodeficient hosts (Rongvaux et al., 2014). This allows researchers to focus on human-specific mechanisms without the added complexity of tissue containing mixed species. Therefore, we encourage researchers to carefully consider the respective strengths and limitations of *in vivo* and *in vitro* models, to select the most appropriate approach based on experimental goals, available resources and biological questions.

## Future opportunities and challenges

Looking ahead, the *in vivo* organoid platform presents an exciting avenue for studying neuroimmune interactions in health and disease, complementing existing models (Schafer et al., 2023). This platform is particularly valuable for identifying determinants of the homeostatic state of microglia and elucidating interactions between mature microglia and the central nervous system environment under physiological and pathological conditions. This platform would also facilitate investigations into mechanisms underlying neurodevelopmental and neurodegenerative conditions that require mature microglia. For instance, key microglial homeostatic signature genes such as *P2RY12* and spalt-like transcription factor 1 (*SALL1*) are implicated in neurological diseases (Lin et al., 2020; Vodopiutz et al., 2013), and their study will benefit from systems in which they are highly expressed at baseline. Xenotransplanted neuroimmune brain organoids could also enable researchers to study how human microglia respond to systemic signals in the host associated with neurodegeneration, diabetes, infection, injury and cancer, both acutely and longitudinally, given the long-term survival of MLCs *in vivo*. The potential behavioral outputs following transplantation also provides promising avenues for future research. Lastly, xenotransplantation holds significant translational value, such as through studies involving graft-mediated regeneration following injury and preclinical drug efficacy testing on human cells (Bellotti et al., 2024).

Despite its advantages, the *in vivo* neuroimmune organoid approach faces important challenges that need to be addressed. These include low-throughput, high experimental variability and species-specific influences on phenotypes. It is worth noting that hPSC-derived organoid technology was initially developed to reduce reliance on animal models, making transplantation into rodent hosts contradictory to this goal. However, given the evidence that host brain environments enhance both MLC and organoid maturation, it is crucial to identify the factors driving these improvements and apply them to *in vitro* models. For instance, the establishment of perfusable organoids *in vitro* could recapitulate some of the benefits of the vascular and nutrient support observed in xenotransplanted models (Zhao et al., 2021). By identifying and incorporating these instructive factors, researchers could enhance the fidelity and functionality of *in vitro* systems, reducing the need for *in vivo* transplantation.

In conclusion, the *in vivo* neuroimmune organoid system marks an important technological development in human microglia research, enabling specific studies of mature, homeostatic microglia in a physiological context. However, we expect this approach to complement, rather than replace, existing *in vitro* platforms. Together, these hPSC-derived platforms expand the investigative toolkit available for studying neuroimmune interactions, providing invaluable insight into normal brain function and disease mechanisms. We look forward to seeing more studies applying these complementary strategies to elucidate the many unknowns of how innate immunity supports human brain health.
